# Roles of Polycomb Complexes in the Reconstruction of 3D Genome Architecture during Preimplantation Embryonic Development

**DOI:** 10.3390/genes13122382

**Published:** 2022-12-16

**Authors:** Longtao Yu, Hengxiang Shen, Xiaowen Lyu

**Affiliations:** 1 State Key Laboratory of Cellular Stress Biology, Fujian Provincial Key Laboratory of Reproductive Health Research, School of Medicine, Faculty of Medicine and Life Sciences, Xiamen University, Xiamen 361102, China; 2 Fujian Provincial Key Laboratory of Organ and Tissue Regeneration, School of Medicine, Faculty of Medicine and Life Sciences, Xiamen University, Xiamen 361102, China

**Keywords:** Polycomb complexes, PRC, ESCs, 3D chromatin conformation, transcription, preimplantation embryo

## Abstract

The appropriate deployment of developmental programs depends on complex genetic information encoded by genomic DNA sequences and their positioning and contacts in the three-dimensional (3D) space within the nucleus. Current studies using novel techniques including, but not limited to, Hi-C, ChIA-PET, and Hi-ChIP reveal that regulatory elements (Res), such as enhancers and promoters, may participate in the precise regulation of expression of tissue-specific genes important for both embryogenesis and organogenesis by recruiting Polycomb Group (PcG) complexes. PcG complexes usually poise the transcription of developmental genes by forming Polycomb bodies to compact poised enhancers and promoters marked by H3K27me3 in the 3D space. Additionally, recent studies have also uncovered their roles in transcriptional activation. To better understand the full complexities in the mechanisms of how PcG complexes regulate transcription and long-range 3D contacts of enhancers and promoters during developmental programs, we outline novel insights regarding PcG-associated dramatic changes in the 3D chromatin conformation in developmental programs of early embryos and naïve-ground-state transitions of pluripotent embryonic stem cells (ESCs), and highlight the distinct roles of unique and common subunits of canonical and non-canonical PcG complexes in shaping genome architectures and transcriptional programs.

## 1. Introduction

The development of multicellular organisms relies on the establishment and maintenance of cellular identities which are precisely regulated by the spatiotemporal-specific expression of tissue-specific genes. To achieve these objectives, various DNA-binding transcription factors (TFs) bind different regions of the genome to guide the recruitment and activity of RNA polymerase II (Pol II). Recently, with the inventions and improvements in chromatin conformation capture technologies such as Hi-C, Micro-C, Hi-ChIP, and split-pool recognition of interactions by tag extension (SPRITE), it has been realized that 3D organization of the genome is emerging as a major regulator of gene expression programs [1,2,3,4,5]. Firstly, chromosomes are not randomly spread within the interphase nucleus, but instead are hierarchically organized into multiple layers of 3D structures such as loops, TADs and compartments to bring linearly scattered genomic regions of similar epigenetic features into proximity in the 3D space [4,6,7]. Notably, genomic loci with high chromatin accessibility and transcription are more inclined to lie in the internal section of the nucleus and form active compartments, while those with low chromatin accessibility and transcription mostly lie in the periphery of the nucleus to form inactive compartments featured with highly compacted chromatin [8,9]. At the sub-Mb scale, chromatin can be organized into continuous segments, typically named topologically associating domains (TADs), within which chromatin epigenetic states and transcription levels of REs change more synchronously [10]. In addition to TADs, other continuous chromatin segments, such as lamin-associated domains (LADs), nucleolus-associated domains (NADs), and Polycomb-associated domains (PADs), are also reported to occur within specific regions of the genome and physically locate within certain areas of the nucleus [11,12,13,14]. Recent studies indicated that TADs mainly arise from loop extrusion of chromatin by cohesin, while other continuous segments such as LADs, NADs, and PADs usually form through other mechanisms. Accumulating evidence indicates that PcG proteins are also important epigenetic factors that could influence the 3D conformation of chromatin, eventually underpinning their gene expression regulatory functions [15,16,17]. PcG complexes were first identified in Drosophila melanogaster, acting as an inhibitor of the expression of homeotic (*HOX*) genes required for body plan specification [18,19,20,21]. Subsequent biochemical analyses showed that PcG complexes also have important roles in controlling unique gene expression programs and chromatin conformation throughout mammalian development [22,23]. PcG complexes can be roughly classified into two major groups, PRC1 and PRC2, based on their affiliated protein subunits. Although these two groups of PcG complexes share many common targets in the genome, they are quite different in function [24]. PRC1 is mainly responsible for generating mono-ubiquitination of histone H2A at Lys119 (H2AK119ub1), while PRC2 is mainly responsible for the mono-, di-, and tri-methylation of histone H3 at Lys27 (H3K27me1, H3K27me2 and H3K27me3, respectively) [23,25,26]. Although PcG complexes were originally discovered in Drosophila, they perform both conserved and unique functions among different species, especially in mammals, considering the complexity of growth and development. With all this in mind, we will focus on understanding how mammalian PcG are targeted to specific regions of the genome and spread to other loci to ultimately regulate gene expression. In the first part of this review, we will introduce the diverse PcG complexes and their corresponding enzymatic activities. Then, we will focus on PcG-dependent changes in 3D chromatin organization during early embryonic development, and stem cell pluripotency maintenance and withdrawal. Finally, we will systematically highlight some possible mechanisms by which PcG complexes perform their respective functions during the above cellular process.

## 2. The Wide Repertoire of PRC

Although PcG are evolutionarily conserved multiprotein complexes, they still function differently in mammals than in Drosophila. Through their respective auxiliary proteins, PcG proteins are recruited to specific genomic regions, thereby differently catalyzing these corresponding regions relying on their catalytic cores; finally, they contribute to the regulation of key developmental genes, determining cell identities via the arrangement of 3D genome organization. Considering the above diverse functions, understanding the composition of different PcG complexes and their respective activities, such as enzymatic activities, is a prerequisite for elucidating the full complexity of mammalian PcG in gene regulation during development.

### 2.1. The Composition and Functions of PRC1

**Figure 1:**
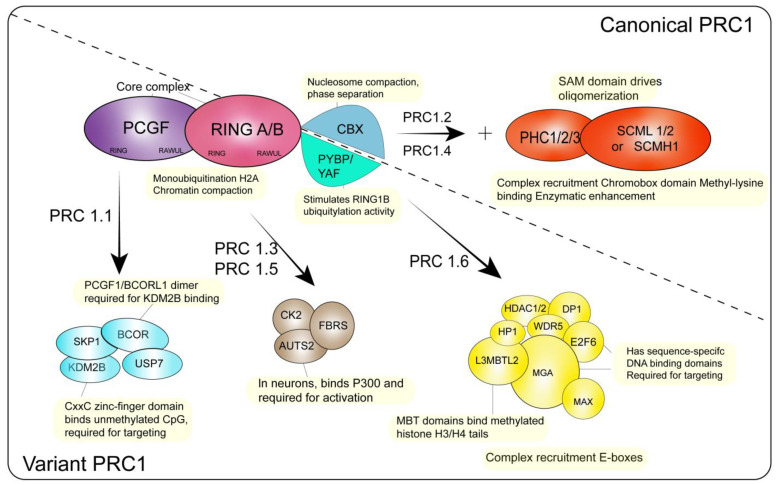
Comparisons of different groups of PRC1 complexes. Illustration of core and accessory subunits composing canonical and variant PRC1 complexes.

The core components of PRC1 include RING1A, or its paralogue RING1B, and Polycomb group RING finger (PCGF) proteins which have similar domain structures [27] (Figure 1). The N-terminal RING domain is responsible for dimerizing PCGF and RING1A/1B, facilitating their interaction with an E2 conjugating enzyme. This way, RING1A/1B monoubiquitylates H2AK119ub1 at promoters and across the genome to repress the transcription of target genes [25,26]. The RAWUL domain is responsible for binding a range of auxiliary proteins (including CBX/RYBP and PCGF-related auxiliary subunits), thus playing a vital role in recognizing specific sites in the genome and regulating the catalytic activity [28,29,30]. According to different auxiliary subunits, PRC1 can be further divided into canonical PRC1 (cPRC1) and variant PRC1 (vPRC1). cPRC1 complexes include one of five chromodomain-containing paralogues, which are mainly responsible for recognizing the H3K27me3 locus across the genome [22,23,31]. cPRC1 contains different CBX subunits at different stages of embryonic development. Compared with CBX7, CBX2 contains an intrinsically disordered region, which can neutralize the positive charge of DNA and bring them together, therefore promoting chromatin compaction [32,33]. In this respect, PcG domains of ESCs are more easily bound by TFs. CPRC1 also includes the polyhomeotic (PHC) subunit (PHC1, PHC2 or PHC3), and the SAM domain of these accessory proteins drives the oligomerization of PRC to promote phase separation [34,35,36].

As opposed to cPRC1, vPRC1 complexes can assemble around any of the six PCGF proteins and different auxiliary subunits depending on the PCGF component present in the complex. Besides the PCGF component, binding with the vPRC1-specific auxiliary subunit RING1 and YY1-binding protein (RYBP) and its paralogue YAF2 can also dramatically stimulate E3 ligase activity and influence the catalytic activity of RING1A/1B [27,37]. By contrast, as CBX complexes have a far less pronounced above effect, the catalytic activity of the cPRC1 complexes is thus far less active than that of vPRC1 complexes [38,39]. According to the types of PCGF proteins contained, vPRC1 complexes can be further divided into vPRC1.1 (containing PCGF1), vPRC1.3/1.5 (containing PCGF3/5), and vPRC1.6 (containing PCGF6). Different accessory proteins enable the corresponding vPRC1 to recognize specific sites across the genome. KDM2B mainly binds to the unmethylated CpG island, whereas AUTS2 mainly binds to P300, promoting the activation of target genes; L3MBL2 mainly binds to the tail of methylated H3/H4 [40,41,42,43].

### 2.2. The Composition and Functions of PRC2

**Figure 2:**
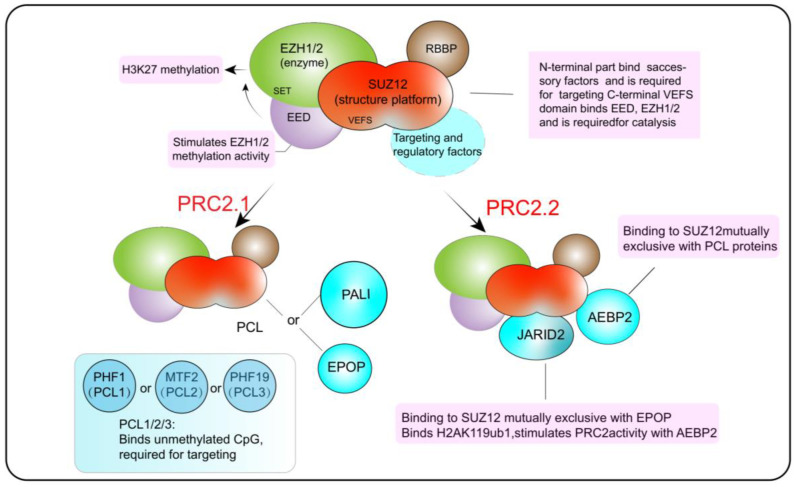
Comparisons of different groups of PRC2 complexes. Illustration of core and accessory subunits composing canonical and variant PRC2 complexes.

The core catalytic proteins complexes of PRC2 include retinoblastoma-binding protein 4 (RBBP4) or retinoblastoma-binding protein 7 (RBBP7), embryonic ectoderm development (EED), enhancer of zeste 2 (EZH2) or its paralogue enhancer of zeste 1 (EZH1), and suppressor of zeste 12 (SUZ12) (Figure 2). SUZ12 is equivalent to a structural protein, and EED is responsible for regulating the catalytic activity of EZH2, which engages with nucleosomal DNA and catalyzes the mono-, di-, and tri-methylation of H3K27 (H3K27me1, H3K27me2 and, H3K27me3) [44,45,46]. According to the different auxiliary proteins taking charge of recognizing specific sites across the genome, PRC2 can be further divided into PRC2.1 and PRC2.2. The PCL protein in PRC2.1 mainly binds to the non-methylated CpG site, while AEBP2 and JARID2 in PRC2.2 mainly bind to the H2AK119ub1 site, stimulating H3K27 methylation [34,47,48,49].

## 3. Roles of PcG Complexes in 3D Genome Organization during Embryonic Development

### 3.1. Roles of PcG Complexes in the Dissolution of 3D Chromatin Organization before Fertilization

#### 3.1.1. PcG-Associated 3D Chromatin Organization in Sperm

**Figure 3:**
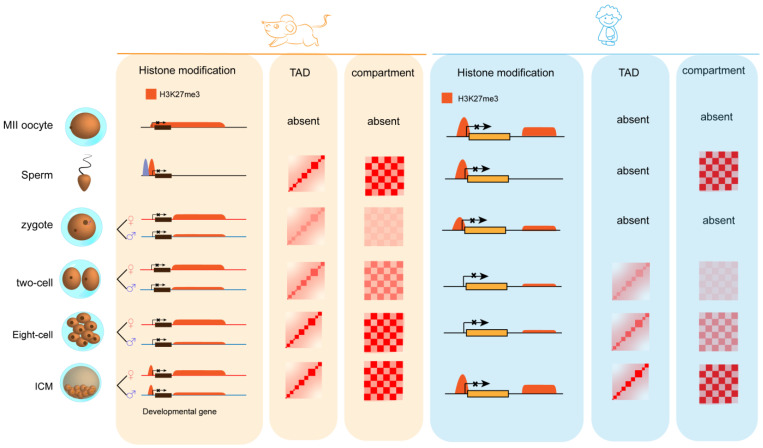
Gradual reconstruction of TADs and compartments during early embryonic development. Gradual reprogramming of epigenetic states and reconstruction of TADs and compartments during early embryonic development in a mouse (**left** panel). Gradual reprogramming of epigenetic states and reconstruction of TADs and compartments during early embryonic development in a human (**right** panel).

The sperm genome is wrapped by a large amount of protamine into highly condensed chromatin, which impedes the recruitments of TFs and eventually switches off global transcriptions in oocytes [50]. Recent studies show that, like most mammalian cells, such as mouse ESCs (mESCs) or somatic cells, sperm still have TADs and compartments because chromatin architecture proteins including CTCF and cohesins are still loaded on the genome [51] (Figure 3). However, it should be noted that, compared with ESCs, sperm contains some sperm-specific characteristics, such as more long-distance interactions of chromatin [52,53]. Limited by the previous resolution of Hi-C technology, up to now, the relationship between chromatin epigenetic states and chromatin 3D contacts of the sperm genome has not been clarified. However, more and more studies have shown that sperm-specific long-distance interactions may result from H3K27me3 modifications on histone tails [54]. H3K27me3 is usually deposited by PRC2 in the promoters of developmental genes, while PRC1 can recognize H3K27me3-labeled promoters. The CBX and PHC subunits mentioned above can help PRC1 to compact local and distal chromatin by mediating long-range interactions. Although most histones are replaced by protamine during spermatogenesis in mammals, H3K27me3 can still be detected on the residual histones located at promoters of many developmental genes. Therefore, it is possible that H3K27me3 plays an important role in maintaining sperm-specific long-distance interactions. Correspondingly, KMT2B, has been shown to be necessary for spermatogenesis and embryonic development [55,56]. Similarly, knockout of H3K27me3 methyltransferases EZH1, and EZH2 will lead to meiosis block of sperm [57]. The above studies indicate that PcG complexes play important roles in spermatogenesis by mediating extremely long-distance chromatin contacts in the sperm genome.

#### 3.1.2. PcG-Associated 3D Chromatin Organization in Oocytes

With the growth and maturation of follicles, oocytes transit from non-surrounded nucleolus (NSN) oocytes to surrounded nucleolus (SN) oocytes, in which chromosomes form a rim surrounding the nucleolus, accompanied by genome-wide transcription silencing and chromatin compaction [57]. This transformation is mainly important for maintaining the integrity of chromosomes during meiosis [58]. Although loops and TADs exist throughout all stages of germinal vesicle (GV) oocytes, they are still different between stages, in that NSN oocytes display more short-range (<400 kb) contacts, but weaker loops and TADs than SN oocytes [58]. Accordingly, a decrease in transcription is observed in SN oocytes that have more compacted chromatin [59]. Additionally, it has been reported that transcriptional elongation mediated by RNA polymerases is more active in NSN oocytes than in SN oocytes. Interestingly, the reorganization of the 3D chromatin conformation in GV oocytes is accompanied by changes in the distribution of H3K27me3 on chromatin. In growing oocytes, especially NSN oocytes, H3K27me3 is not enriched on the promoters of developmental genes, but is deposited in the form of wide peaks within transcriptionally inactive regions and gene deserts [60]. In contrast, SN oocytes gradually lose those wide peaks, and only keep H3K27me3 in a portion of those regions with low levels of transcription [60]. This down-regulation of transcription levels in SN oocytes seems contradictory to the transcriptional repression outcomes of H3K27me3 deposition on chromatin. However, recent studies indicated that PcG complexes that can deposit and read H3K27me3 do not necessarily inhibit transcription, but are instead more involved in chromatin compaction. Furthermore, although the distribution of H3K27me3 in SN oocytes is not as wide as that in NSN oocytes, the enrichment levels of H3K27me3 in the SN phase are higher than those in the NSN phase. Therefore, it is very likely that PcG complexes play an important role in chromatin compaction in oogenesis. Unlike GV oocytes, loops and TADs disappear in metaphase II (MII) oocytes [54,59,60]. Although H3K27me3 is deposited in fewer sites of the genome during this transition, the disappearance of chromatin structures is more due to the arrest of oocyte division in metaphase.

### 3.2. Roles of PcG Complexes in the Dissolution of 3D Chromatin Organization after Fertilization

After returning the karyotypes to diploids after the fusion of male and female pronuclei, embryos continue to cleavage with the recovery of cell cycle progression. This process is accompanied by changes in chromatin 3D conformation, including the allele-specific reprogramming of epigenetic memories of the parental genome. It should be noted that, unlike somatic cells, chromatin 3D organization is gradually established during the cleavage and preimplantation development of embryos. Although male and female pronuclei fuse after fertilization, the chromosomes of the two sets are still partially separated within the nuclear envelope [61,62]. Taking mice as an example, with the gradual replacement of protamine by the maternal proteins, the strong TADs and compartments present in the paternal chromosomes disappear [54]. Although the degrees of compartmentalization in the female genome increase, those in the paternal genome are still higher globally. This difference in 3D chromatin architecture between parents does not disappear until the eight-cell stage [63]. In the late one-cell stage and early two-cell stage, chromatin becomes looser, so that TFs such as DUX can be recruited to activate the transcription of repeated elements such as MERVL [64]. With the activation of the embryonic genome, TADs and compartments are reconstructed in the two-cell stage, and their intensities increase continuously, giving rise to the observation that interactions within the same compartments increase, and the interactions between different compartments decrease [50]. Although the re-establishment of TADs coincides with zygote genome activation (ZGA), there is no obvious relationship between these two events. Indeed, the re-establishment of most TADs is not significantly affected when embryos are treated with transcriptional inhibitors in the zygotic stage, which is also the main difference between mouse and human in the reconstruction of TADs and compartments during early embryonic development [63,65]. The reconstruction window of TADs in human preimplantation embryos also occurs in the eight-cell stage, coinciding with ZGA; however, the reconstruction of TADs is significantly inhibited after the treatment of zygotes with transcription inhibitors. This disruption of TADs in ZGA by transcription inhibition is also observed in other mammals. For example, a recent study using a transcription inhibitor to treat fly Kc167 cells demonstrated that interaction domains are related to transcription [66]. Correspondingly, H3K27me3 also undergoes significant erasures and reconstruction after fertilization. The classical narrow peaks of H3K27me3 usually enriched in the promoter regions of developmental genes in sperm are likely to be rapidly removed after fertilization, while the non-classical H3K27me3 in the distal regions remains until the inner cell mass (ICM) stage, although the enrichment levels are low. It is not until ICM that the promoters of developmental genes begin to deposit H3K27me3, and the deposition levels increase continuously in the subsequent developmental stages [67]. Oocyte-specific, wide, non-canonical (NC) H3K27me3 at the promoters of developmental genes is specifically erased after fertilization, while ncH3K27me3 at distal sites is maintained up to the later stages, as late as ICM [60]. Maternal knockout of the core catalytic subunit RING1A/1B of PRC1 can affect ZGA and cell divisions of embryos, and finally leads to developmental arrest in the two-cell stage [68]. The above studies show that PcG complexes can inhibit differentiation-related genes through PRC1-mediated K119 ubiquitination modification and participate in the reprogramming of chromatin states and reconstruction of the 3D chromatin architecture during development. In addition, H3K27me3 also plays similar roles in the above developmental process. Maternal knockout of Ezh1 and Ezh2 hinders the H3K27me3 re-establishment of mouse preimplantation embryos and eventually leads to abnormal development of embryos after implantation [69]. H3K27m3 can also be deposited on the promoters of ZGA genes together with H3K4me3, and the inhibition of this deposition will induce early activation of ZGA genes [53]. These results indicate that PcG complexes generally play important roles in the development of preimplantation embryos in mammals.

## 4. Regulatory Mechanisms of PcG Complexes

### 4.1. Recognition of Target Sites by Multiple Mechanisms

The ability of PcG complexes to specifically recognize different sequences of the genome is the premise that PcG complexes regulate the transcription levels of target genes by affecting their distal chromatin contacts in the 3D space. Although many studies in mammals and Drosophila melanogaster showed that canonical PcG complexes are usually engaged with the transcription repression of target genes, recently, many variant PcG complexes containing non-canonical accessory subunits have also been implicated in the transcription activation of target genes by binding to their promoters and relative REs [70]. To better understand how distinct PcG complexes regulate the transcription and 3D chromatin contacts of genes, here we list multiple mechanisms through which different PcG complexes recognize their target genes and bring them closer to specific REs in the 3D space to effectively repress or activate their transcription.

#### 4.1.1. Targeting of PcG Complexes by DNA Sequences

PcG complexes, originally found in Drosophila, usually recognize different target genes through their sequence-specific DNA binding accessory proteins [23,71]. Although some orthologues of the above accessory proteins are found in mammals, they are not able to target PcG complexes to chromatin. Recently, more and more accessory proteins of PcG complexes in mammals have been validated to specifically recognize target genes [20,72,73,74,75]. Correspondingly, when ncPRC1.6 binds to specific sites through these accessory proteins, RING1A/1B exercises its K119 ubiquitination function and inhibits the transcription of germ cell-related genes [70,76]. Conditional depletion of PCGF6 subunits in ncPRC1.6 in mESCs leads to the de-repression of these genes, which will affect the growth and fate of cells [77]. In mESCs, ncPRC1.6 can recognize E-box motifs (5′-CACGTG-3′) and longer T-box motifs (5′-TCACACCT-3′) through accessory proteins MGA and MAX, thus recognizing many genome-specific sites including promoters of germ-cell-specific genes [78,79]. Both Myc and Mad as well as MGA can form a dimer with MAX to recognize specific DNA sequences, recruiting transcription coactivators or transcription inhibitors, followed by changing the chromatin structure and ultimately regulating the transcription of target genes [79]. Furthermore, even though the E2F6-DP1 heterodimer can only target ncPRC1.6 to E2F-binding sites when working together with MGA/MAX, it can also promote the targeting of ncPRc1.6 to Myc-and Brachyury-binding sites, which are usually recognized by MGA/MAX [78]. Besides PRC1, PRC2 can also be targeted to specific regions of the genome by its accessory proteins. For example, PRC2.2 can recognize H2AK119ub1 specifically through its accessory protein JARID2, which cooperates with RING1A/1B to modify these target sites with H3K27me3 [80]. In ESCs, JARID2 mainly inhibits the expression of the pluripotent transcription actor Nanog, thus promoting the withdrawal of pluripotency. In *Jarid2* (-/-) ESCs, the expression of Nanog was abnormally high, while the transcription levels of differentiation-related genes such as *Wnt9a, Prickle1, and Fzd2* were down-regulated; injection of *Jarid2* (-/-) ESCs into normal E3.5 blastocysts will lead to the overexpression of Nanog and a significant increase in cell numbers of the inner cell mass (ICM) [81,82]. The above studies show that PRC2.2 can recognize specific DNA sequences through JARID2, thus regulating gene transcription and controlling the cell fates of pluripotent stem cells and early preimplantation embryos.

#### 4.1.2. Targeting of PcG Complexes through Specific TFs

Although other accessory proteins cannot bind chromatin directly in the same way as MGA/MAX and E2F6-DP1, they can bind chromatin indirectly by briefly interacting with other sequence-specific DNA-binding TFs, thus enabling different PRC complexes to methylate or ubiquitinate chromatin at different genomic regions. For example, experiments with highly specific PCGF1-6 antibodies and mESCs depleted of each PCGF protein implied that PCGF3 enables the localization of ncPRC1.3/1.5 to some genomic regions by interacting with TFs such as USF1, USF2, and NRF1 [79]. It should be noted that, unlike other PRC1 complexes, ncPRC1.3/1.5 is mainly associated with chromatin in an active transcriptional state. Consistent with the above study in ESCs, ncPRC1.3/1.5 can bind P300 through its accessory protein AUTS2 in neurons to promote gene transcription [83]. Similarly, cPRC1 complexes can also change their genomic targets by replacing subtypes of their accessory protein REST. It has been suggested that REST-associated proteins can help cPRC1 in repressing genes related to neural differentiation in mESCs. Deletion of the amino-terminal regions of REST leads to decreased and increased accumulation of PRC1 in distal and proximal RE1 elements, respectively, accompanied by the up-regulation of genes related to neural differentiation [84]. Moreover, in differentiated cells, PcG complexes can utilize accessory proteins such as RUNX1 and SNAIL1 to bind specific genomic regions [85,86]. Notably, although accumulating studies in recent decades indicated that PRC binds specific genomic regions either directly or indirectly through approaches including but not limited to those listed above, novel approaches to confining the binding specificities of PRC complexes still need to be further explored to better understand their regulatory mechanisms.

### 4.2. Spreading of PcG Complexes across the Genome

PcG proteins, as one of the essential developmental regulators, partially control the transcription of many tissue-specific TFs mainly involved in cell fate determination during early embryonic development, and the maintenance and withdrawal of stem cell pluripotency. Over the past decades, it has been well known that PcG proteins can poise important developmental genes in transcriptional repressive states by occupying their promoters or enhancers, thus hindering the recruitment and activation of the transcription machinery [87]. Moreover, the observed concentration of PcG complexes in small bodies within the nucleus might come from long-range chromatin contacts of distant PcG-targeted genomic regions, facilitating the synchronous repression of multiple PcG targets in a transient time window during early embryonic development. To better understand how PcG complexes control developmental programs through diverse mechanisms, we will highlight recent findings in 3D genome organization that validate the roles of PcGs in mediating chromatin long-range interactions and focus on how PcGs spread on distant REs across the genome through diverse mechanisms.

#### 4.2.1. Spreading of PcG Complexes by Local Folding

**Figure 4:**
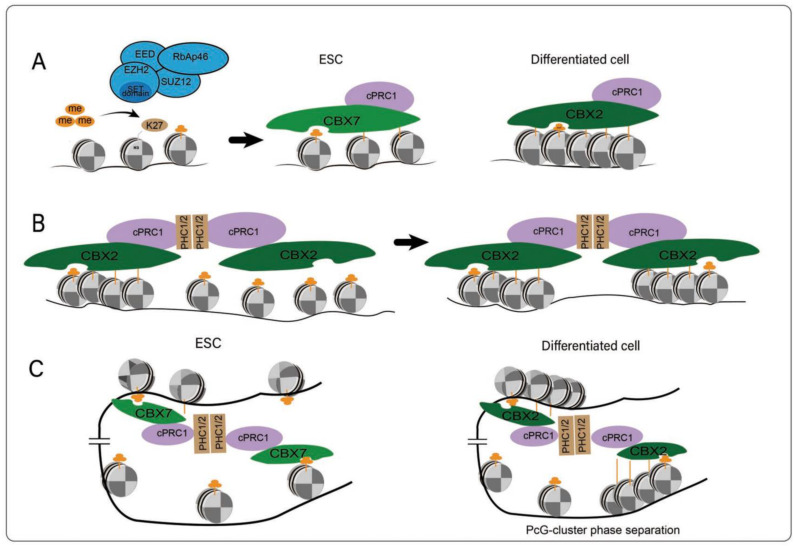
Mechanisms of establishment of long-distance chromatin interactions between distant genomic loci during differentiation of ESCs. (**A**) Depiction of PRC1 containing CBX7 in ESCs and CBX2 in differentiated cells. (**B**) Local compaction of chromatin by CBX2 in differentiated cells. (**C**) Spreading of PRC1 through long-distance contacts between distant genomic loci mediated by CBX7 and CBX2 subunits in ESCs and differentiated cells, respectively.

One of the most important functions of PcG complexes is to poise the transcription of key developmental genes such as *HOX* clusters by condensing the nucleosome arrays on their promoters to compact local chromatin. The ability of PcG complexes to compact local chromatin was first identified in Drosophila melanogaster and later observed in mammals. Once PcG complexes are loaded on chromatin, the accessibility of chromatin will decrease, thus impeding the accumulation of TFs and eventually inhibiting the transcription of target genes [36,88]. This conserved chromatin compaction function of PcG complexes between mammals and Drosophila is mainly achieved by CBX2, a core subunit of cPRC1, which contains a compaction region required for the compaction of adjacent nucleosomes. CBX2 contains a large amount of low-complexity disordered regions (LCDRs), in which the positive charges neutralize the negative charges in DNA, resulting in decreased repulsion between DNA, bringing them together and eventually achieving the compression of local chromatin [32,89] (Figure 4). At first, PRC2 complexes bind a specific genomic locus to write H3K27me3 on local chromatin, which can be recognized by cPRC1 accessory proteins such as PCGF2/4 [80,81]. Once cPRC1 binds chromatin, the chromatin can be condensed by CBX2. Correspondingly, the knockdown of EZH2 leads to a decrease in the level of genome-wide H3K27me3 deposition and a significant decrease in the number of PcG bodies, which is likely due to the decreased amount of CBX recruited [90]. However, it is important to note that PcG complexes containing various CBX proteins are differentially expressed during embryogenesis, of which only CBX2 contains a positively charged LCDR [87]. In other words, PcG-occupied chromatin can be different in chromatin accessibility depending on the CBX proteins that PcG complexes contain. Correspondingly, ESCs are more inclined to express CBX7 lacking an LCDR domain; thus, chromatin accessibility is higher and more TFs can bind to chromatin to promote gene expression. Although CBX7 does not have the capacity to condense chromatin, these PRC complexes containing CBX7 can still interconnect with each other through the head-to-tail polymerization of PHC, which is also closely related to H3K27me3. This seems to contradict previous studies showing that the conserved chromodomain (CD) of CBX7 exhibits a preference for H3K9me3 [91]. In fact, this phenomenon is mainly found in fruit flies. Using live-cell single-molecule tracking (SMT) and genetic engineering techniques in mESCs, studies have shown that H3K27me3 is essential for CBX7 and CBX8 to target chromatin, but not very important for CBX2, CBX4, and CBX6. CBX7 cannot stably bind chromatin merely by the interaction between its CD and H3K27me3 [91]. These studies indicate that PcG complexes could have diverse approaches to bind and condense chromatin.

#### 4.2.2. Long-Range Interactions Mediated by PcG Complexes

Of tens of thousands of DNA loops in mESCs, about 4000 loops, spanning tens to hundreds of megabases across multiple TADs, form between H3K27me3-enriched chromatin loci. Additionally, anchors of most of these loops are enriched with Polycomb nucleation points that overlap with many key developmental genes, such as *HOX* clusters. Removal of Ring1A/B alters the modification of H3K27me3 and disrupts the spatial interactions of *HOX*, leading to the ectopic activation of *HOX* genes [92]. It can be interpreted that PcG complexes can tune the transcription of those genes by establishing long-range looping interactions between promoters and enhancers, to facilitate the spreading of PcG complexes across the genome. As a remote regulatory element, enhancers mainly contain a characteristic mono-methylation of histone H3 lysine 4 (H3K4me1), and can be further divided into three categories according to the histone modification states on them: active enhancers (H3K27me3-, H3K27ac+), poised enhancers (PEs) (H3K27me3+, H3K27ac-) and primed enhancers (H3K27me3-, H3K27ac-). By recognizing H3K27me on PEs, PcG complexes repress those developmentally critical target genes by establishing physical PE-promoter contacts in undifferentiated stem cells, even prior to differentiation [93]. This spreading mechanism enables target genes to respond immediately to differentiation stimulus signals. Accordingly, H3K27me3-decorated chromatin regions are usually found within Hi-C compartment A, which are enriched with active histone modifications, such as H3K27ac and actively transcribed genes [94]. In agreement, deletions of loop anchors disrupt PcG-dependent spatial interactions and eventually lead to ectopic activation of their target genes. Considering that knocking out EED also results in similar outcomes, PRC2 may play an important role in establishing long-range chromatin interactions [92,95]. PRC2 can locally increase H3K27me3 and, consequently, promote the binding of PRC1 at target sites. Additionally, mutations of the sterile α motif (SAM) domain of the PRC1 subunits also disrupt these long-range interactions [34]. The above shreds of evidence suggest that SAM domains seem to also be important for the establishment of PRC1- and PRC2-dependent long-range contacts to encourage the spreading of PcG complexes across the genome.

## 5. Conclusions

The 3D conformation of chromatin plays an important role in the transcriptional regulation of pluripotency and development-related genes. The PcG, one of the chromatin-associated complexes originally discovered in D. melanogaster, has also been shown to be involved in these processes. Due to the lack of RYBP and other accessory proteins to stimulate the catalytic activity of RING1A/1B, the activity of PRC1 in depositing H2AK119ub is quite low. Conversely, as CBXs contain LCDRs, whose positive charges neutralize the negative charges of DNA, PRC1 has a great effect on chromatin condensation. Since ESCs preferentially express CBX7, which lacks LCDRs, their chromatin is more accessible, allowing for strong occupancy by TFs to eventually promote the transcription of target genes. Recent studies have shown that PcG complexes can also promote transcription in some cases, which mainly depends on their accessory proteins and the chromatin environment of their targets. AUTS2, the component of vPRC1.3/1.5, can bind P300 to promote the transcription of target genes in neurons. Consistently, in ESCs, PcG-dependent domains are also found to be located in the active compartment, with high chromatin accessibility and H3K27ac. These PcG domains enable genes to quickly respond to external differentiation signals. In addition to transcriptional activation, PcG complexes exert more functions of transcriptional inhibition on genes. In mESCs, PRC2.2 inhibits Nanog expression and promotes differentiation through its accessory protein JARID2 [81]. However, in naïve human ESCs (hESCs), PcG complexes keep the promoters of genes coding TFs involved in the differentiation towards cell fates, such as the trophectoderm and mesoderm, in a bivalent state to shield them from premature transcriptional activation. Inhibition of PRC2 using an EZH2 inhibitor forces naïve hESCs to differentiate into either trophectoderm or mesoderm lineages [96]. The above findings indicate that during the process of pluripotency maintenance in ESCs, further exploration into how PcG complexes are recruited to these sites and which TFs they can interact with, as well as the specific mechanisms of how PcG complexes are erased in the process of differentiation, is needed. Additionally, PRC2 is mainly responsible for the deposition of H3K27me3 at target sites, and promotes the recognition of these sites by PRC1 to establish long-distance contacts, such as enhancer–promoter contacts and promoter–promoter contacts, between different sites of the genome. However, the contributions of PRC2 to these contacts in ESCs need to be further investigated. Furthermore, considering that PRC1 can achieve diverse functions depending on its accessory proteins, further detailed experiments are needed to investigate the contribution of different components of PRC1 to the construction of 3D chromatin conformation during cellular processes of embryonic development.

## Data Availability

Not applicable.
